# Source characteristics and tectonic impacts on cluster debris flow hazards assessment: Evidence from the Tieba River Basin, NE Qinghai-Tibet Plateau

**DOI:** 10.1371/journal.pone.0355619

**Published:** 2026-08-13

**Authors:** Lingzhi Xiang, Na Shen, Xiangyang Yang, Xiaolong Li, Weimin Yang, Weijia Fan

**Affiliations:** 1 National Inland Waterway Regulation Engineering Technology Research Center of Chongqing Jiaotong University, Key Laboratory of Geological Hazards Mitigation of Mountainous Highway and Waterway, Chongqing Jiaotong University, Chongqing, China; 2 Institute of Geomechanics, Chinese Academy of Geological Sciences, Beijing, China; Sathyambama Institute of Science and Technology: Sathyabama Institute of Science and Technology (Deemed to be University), INDIA

## Abstract

A clustered debris flow event triggered by extreme rainfall on August 17, 2020 in the Tieba River basin has been investigated. Unlike typical debris flow-prone areas, the absence of landslide-derived source materials was notably observed, indicating a unique genetic mechanism. To address this, a novel approach utilizing remote sensing and GIS has been proposed, focusing on the characteristics of solid material sources and structural impacts. To systematically analyze the phenomenon, debris flow gullies and key geomorphological parameters were initially identified through multi-source remote sensing and field surveys. The analysis revealed two critical genetic factors: (1) debris flow scale is derived from source characteristics, and (2) tectonic regulation of drainage patterns (tectonic impacts). Subsequently, solid material sources were classified into three groups, and the volume of loose solid materials (V_s_) was estimated for each catchment. Then, the rainstorm-flood method was applied to 24 selected debris flow cases to calculate total discharge (Qₜ), which represents the debris flow scale under specific rainfall frequencies. Through regression analysis, a relationship between Qₜ and Vₛ was derived, followed by the development of predictive models for the deposition extent (width B and length L). This step not only linked sediment supply to flow dynamics but also enabled preliminary hazard zone delineation. To further incorporate tectonic influences, the angle (T) between debris flow channels and regional stress fields was quantified. By integrating Vₛ, T, and six geomorphic factors (F, Re, D_s_, J, S, HI), a comprehensive hazard assessment system was constructed, bridging source materials, tectonic controls, and geomorphic conditions. Finally, an integrated model was developed using information entropy theory. The results indicate that 16 gullies have a medium to high hazard level, accounting for 66.7% of the total 24 gullies, which is largely consistent with the actual disaster situation. This study establishes a quantitative framework for debris flow hazard assessment in landslide-derived sediment-scarce, tectonically active regions, providing actionable insights for risk management.

## 1. Introduction

Since August 2020, three consecutive rainstorm events have struck southeastern Gansu,each causing widespread impact and severe damage. These extreme rainfall events triggered large-scale debris flow disasters across the region(https://www.sohu.com/a/431647095_120207621). Based on disaster records and field investigations, this marks the first recurrence of such extensive debris flows in nearly 40 years—since 1984. In the Tieba River Basin of Zhouqu County (104°28′ - 104°40′E, 33°13′ - 33°26′N), this was the first recorded debris flow disaster in history. As a result, roads, farmland, and local residents’ houses within the basin experienced unprecedented destruction.

**Fig 1:**
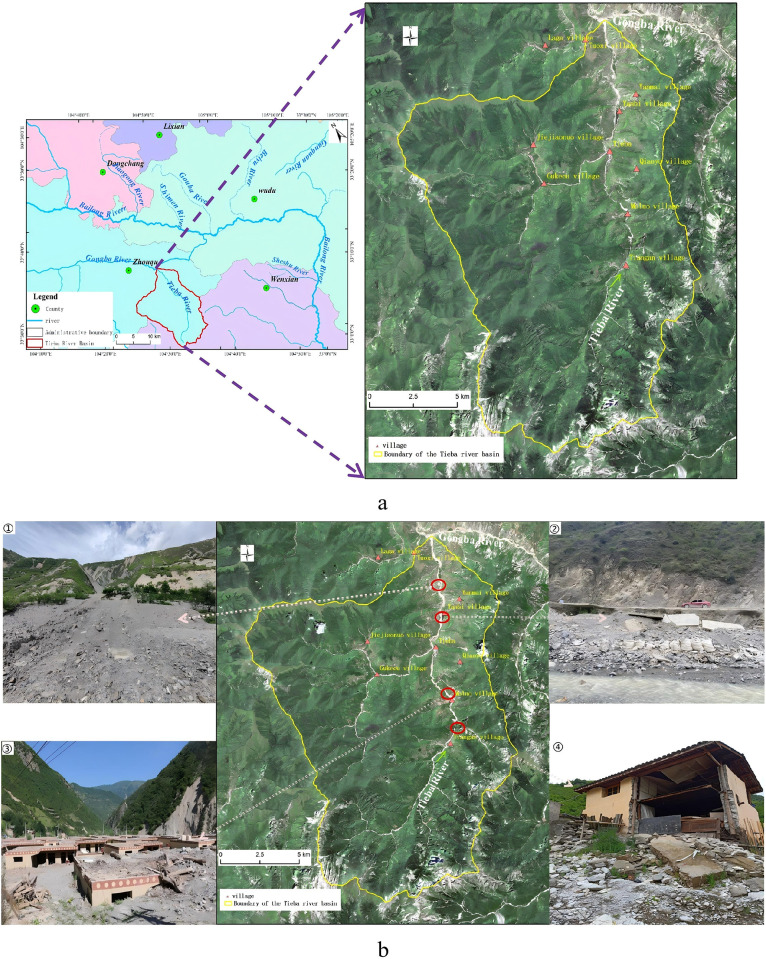
Images and damages before and after the August 17, 2020 debris flow in the Tieba River Basin (The images are sourced from ESA Sentinel-2 data with a resolution of 10 m. The Sentinel-2 imagery used in this figure is publicly available from the Copernicus Open Access Hub (https://scihub.copernicus.eu). Figure a is a schematic diagram of the study area’s location and the pre-disaster image, taken on August 9, 2020. Figure b is the post-disaster image, taken on August 24, 2020. The disaster affected points in Figure b are as follows: ① The debris flow in Yanbi Village washed away the road; ② The Fangzu Gully debris flow in Tuoxi Village buried farmland; ③ The debris flow in Moluo Village buried newly built houses; ④ The debris flow in Tiangan Village washed away the houses at the gully mouth.).

By comparing multi-source remote sensing imagery taken before and after the event and conducting on-site field surveys, it was found that prior to the disaster, vegetation coverage in the area was dense, with virtually no history of debris flow activity (Fig 1a). Post-disaster investigations further revealed an absence of large-scale landslides within the basin that could have supplied debris material. Despite this, the debris flow volume was exceptionally large (Fig 1b), blocking river channels, burying houses and farmland, and severely damaging infrastructure (Fig 1b).

These findings highlight a critical challenge: for small basins with highly unique geological and geomorphic conditions—where no prior debris flow activity has occurred, and extreme rainfall can trigger catastrophic events without the presence of typical landslide-derived debris sources. Therefore, it is urgently necessary to develop predictive capabilities for assessing potential debris flow scales and to conduct comprehensive hazard analyses in such high-risk areas.

With the growing influence of climate change and human activities, debris flow disasters are becoming more frequent and severe, impacting socio-economic and environmental systems. In recent years, significant progress has been made in debris flow hazard assessment at home and abroad, mainly in four key aspects.

First, in debris flow hazard assessment model development, current evaluation models include empirical, physical, and numerical simulation models. Empirical models take a regional view, using historical data of past debris flow events to explore debris flow characteristics, properties, formation conditions, and recurrence frequencies. They select hazard-inducing factors and use multi-factor analytical methods like AHP, fuzzy comprehensive evaluation, and grey system theory to assess regional debris flow hazards, mainly focusing on debris flow watershed characteristic parameters [1–3]. However, their results often only show individual gully hazard levels and can’t precisely demarcate potential impact areas. In contrast, physical models use experimental and statistical methods to establish relationships between debris flow characteristics (e.g., total volume, and velocity) and deposition range to identify at-risk areas. They simulate and predict debris flow behavior based on mechanical principles such as fluid dynamics and particle movement theories [4]. Although accurate, they rely heavily on detailed geological, hydrological, and meteorological data. Numerical simulation models use high-precision topographic, geological, and meteorological data for hazard zoning through rheological modeling and numerical simulation techniques like FEM and FDM [5]. They can simulate debris flow initiation mechanisms and flow paths, enabling dynamic three-dimensional simulations suitable for hazard prediction. Recent progress in this field has focused on improving fluid behavior modeling using theories related to flood, two-phase flow, or granular flow.

The second crucial aspect of debris flow hazard assessment is the integration of multi-source data and big data utilization. With the rapid development of remote sensing (e.g., LiDAR, InSAR), UAV technology, and IoT sensors, high-precision, multi-dimensional data for hazard evaluation can be obtained [6, 7]. These technologies enhance prediction accuracy and real-time monitoring by automatically identifying complex contributing factors.

Thirdly, due to global warming and more frequent extreme weather events, debris flows have become more frequent and severe, particularly in mountainous regions with high precipitation. Numerous studies have concentrated on the impacts of climate change, incorporating factors such as rainfall intensity, frequency, and geological variations into hazard prediction models [8]. Climate-induced alterations in precipitation patterns have a direct impact on the occurrence and severity of debris flows. An increasing number of studies are examining the long-term trends of debris flow hazards under the scenario of global warming, incorporating climate and hydrological models into predictive frameworks [9, 10].

Fourthly, considering that debris flows are affected by a wide variety of geographical factors, hazard assessment methods demonstrate substantial regional disparities. For example, in mountainous, high-altitude areas, debris flows are closely correlated with triggering factors such as precipitation, snowmelt, and seismic events [11]. Recent research endeavors have put forward region-specific evaluation frameworks, emphasizing the establishment of customized indicators and models [12]. For instance, in China’s Wenchuan earthquake zone, hazard assessments have considered seismic activity and rainfall triggers, leading to the development of specialized models that offer valuable perspectives for early warning systems in similar seismically active regions [13].

In summary, research on debris flow hazard assessment is progressing toward a higher degree of greater refinement, intelligence, and regional specialization. However, a multitude of challenges still remain. This is mainly attributed to the escalating frequency of extreme weather events and environmental alterations, particularly in regions where data availability is restricted and historical records are incomplete. Moreover, the applicability of existing models exhibits substantial variation across diverse geographical regions, which requires further validation and enhancement.

The objectives of this study are as follows:

(1) To formulate a method for quantifying potential material sources and the impact of tectonic activities in small mountainous watersheds featuring dense vegetation, an absence of historical clustered debris flow incidents, and substantial tectonic and rainfall influences.
(2) To construct a prediction methodology for debris flow hazard zones through the integration of geomorphic parameters and the quantification of solid material sources.
(3) To develop a comprehensive hazard assessment model for small mountainous watersheds that can provide a valuable reference for disaster prevention and mitigation endeavors in comparable regions.

The paper is structured as follows

In Section 2, the study site and the disaster are initially presented. In Section 3, the primary methods employed are elaborated. In Section 4, the findings of this study, encompassing key parameters as well as the prediction and severity of debris flow hazards, are presented. In Section 5, the limitations of this paper and future research orientations are deliberated. In Section 6, the conclusions are derived.

## 2. Study site and disaster

**Fig 2:**
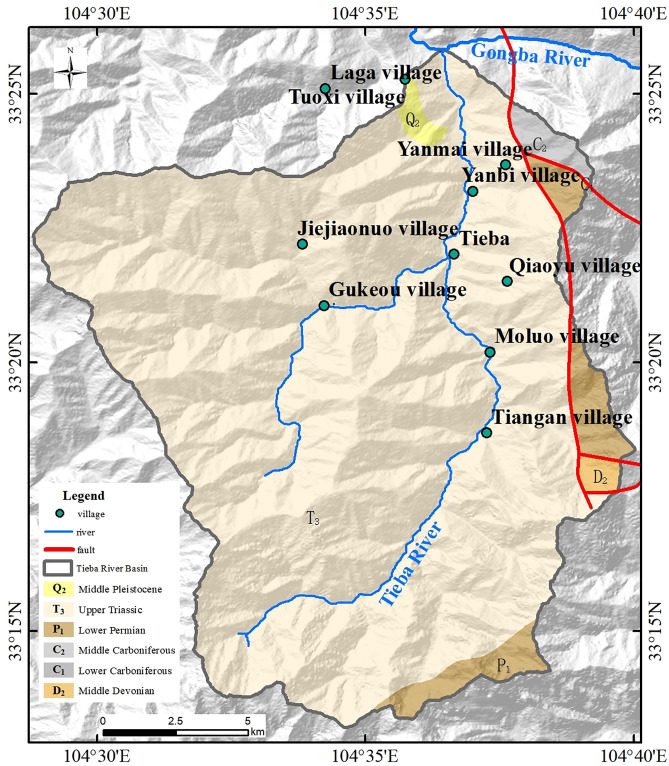
Lithology map of the strata in the Tieba River basin (shaded relief map of the study area derived from ALOS 12.5 m DEM data were obtained from the Geospatial Data Cloud (http://www.gscloud.cn)).

The Tieba River, a primary order tributary of the Gongba River and a second order tributary of the Bailong River, is situated on the right bank of the Bailong River and is a component of the Jialing River system within the Yangtze River basin. It originated from Tieba Village in Qugaona Town, Zhouqu County, Gansu Province, flowing from south to north and converging with the Gongba River at the Dingzi River estuary. This river has a length of 25.6 kilometers, a vertical drop of 1,100 meters, and a riverbed gradient of 34.6‰. The geographical coordinates of the basin are presented in Fig 2.

The lithology of the strata within the study area is predominantly characterized by gray to light gray-green medium-thick-bedded massive sandstone, siltstone, and slate from the Boyuhe Formation of the Upper Triassic (T_3b_), interspersed with a small quantity of thin-layered limestone. Additionally, there are minor occurrences of thin-bedded and lenticular argillaceous limestone and brecciated clastic limestone from the Heihe Formation of the Lower Permian (P_1h_), medium-thick bedded limestone, sandstone, and mudstone with alternating hard and soft layers from the Middle–Upper Carboniferous (C_2 + 3_), as well as gray thin-to-thick bedded limestone and argillaceous limestone from the Sanhekou Formation (D_2s_) of the Middle Devonian (as shown in Fig 2).

Influenced by the temperate monsoon climate, precipitation in this area is concentrated mainly from May to September. The annual precipitation varies between 445 mm and 916 mm, and the average annual temperature is 12.5°C.

Since August 6, 2020, Zhouqu County has experienced four consecutive intense precipitation events, which gave rise to catastrophic flash floods and debris flow disasters. As of 17:00 on August 17, the cumulative precipitation had reached 257.5 mm. The peak flow at the Zhouqu Station of the Bailong River escalated to 880 m^3^/s, exceeding the 20-year flood standard. Meanwhile, the peak flow of the Gongba River exceeded 610 m^3^/s, significantly surpassing the 100-year flood standard.

**Fig 3:**
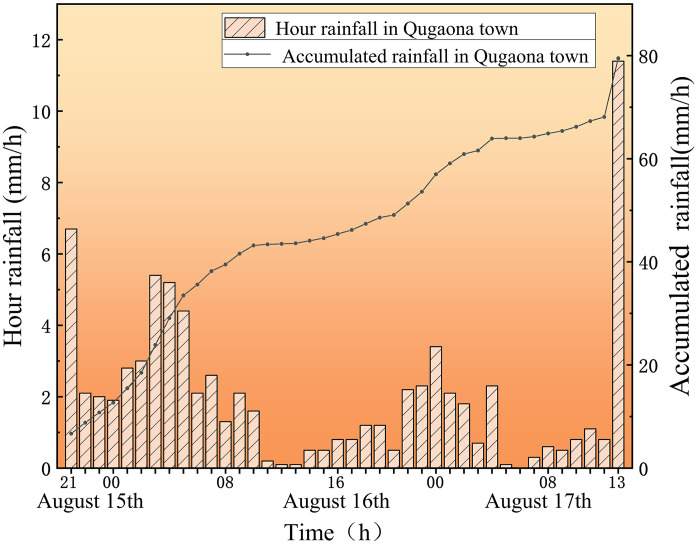
The rainfall process in the Tieba river area on August 17, 2020.

Specifically, within the Tieba River Basin, a tributary region, from 21:00 on August 15–13:00 on August 17, 2020, Qugaona Town in Zhouqu County recorded a cumulative precipitation of 79.5 mm. The primary precipitation period was concentrated between 08:00 and 13:00 on August 17. Notably, from 12:00–13:00 on August 17, the maximum hourly precipitation reached 11.4 mm (as shown in Fig 3).

**Fig 4:**
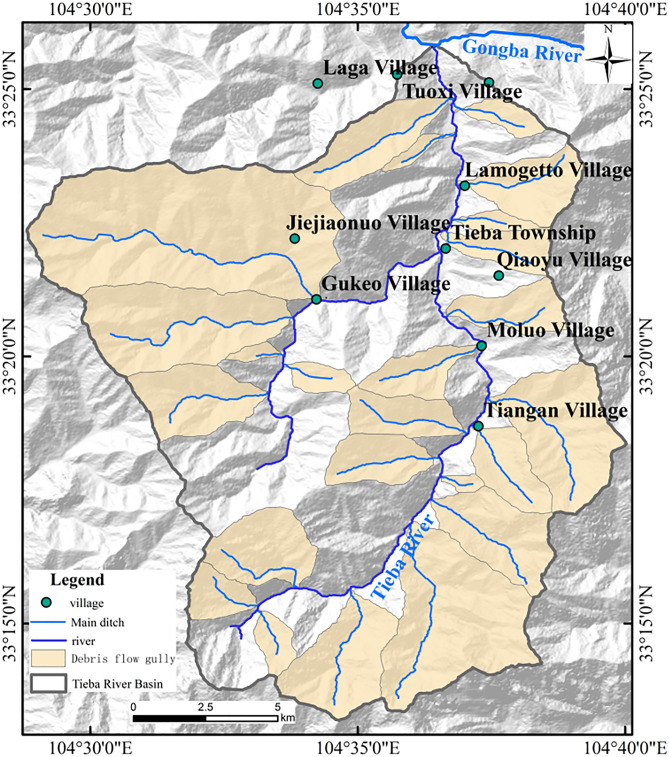
Distribution map of the debris flow in the Tieba River Basin on august 17^th^,2020 (Shaded relief map of the study area derived from ALOS 12.5 m DEM data were obtained from the Geospatial Data Cloud (http://www.gscloud.cn)).

The heavy rainfall triggered mountain torrents and debris flows in 24 primary and tributary gullies of the Tieba River (Fig 4). The alluvial debris flow had an approximate volume of 22,570,000 m^3^, leading to an average riverbed elevation increase of over 2 meters. This natural disaster inflicted severe damage on diverse facilities, such as residential buildings, farmlands, highways, water conservancy projects, power supply systems, and communication networks. The cumulative economic losses reached 1.463 billion RMB.

**Fig 5:**
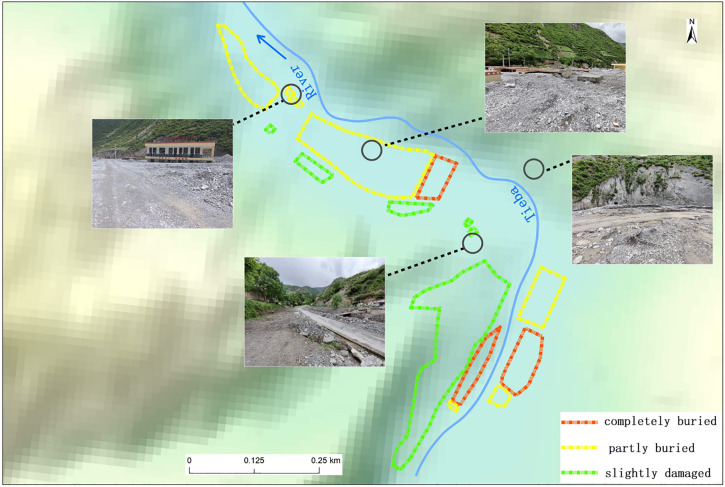
The damages caused by the debris flow in Moluo gully, located on the left bank of the Tieba River (shaded relief map of the study area derived from ALOS 12.5 m DEM data were obtained from the Geospatial Data Cloud (http://www.gscloud.cn)).

Among these disasters, a large scale debris flow took place in Moluo Gully on August 17, 2020. After surging out, the debris flow merged with that from Tieba Gully, inundating newly built houses, farmland, roads, and other infrastructure in the new rural area of Xizhou Group downstream (Fig 5).

## 3. Method

This paper utilizes a variety of essential data, including field investigation data (encompassing debris flow disaster surveys, sampling, and experimental analyses), topographic datasets (ALOS-DEM with a resolution of 12.5 meters), stratigraphic lithology and geological structure materials (1:250,000 scale geological map), hourly rainfall monitoring records (provided by the Zhouqu Meteorological Bureau), and pre- and post-disaster remote sensing imagery (Sentinel-2 remote sensing images with a 10-meter resolution).

Through field investigations and remote sensing analysis, a comprehensive inventory of debris flows was compiled for the 8.17 rainstorm. Subsequently, the fundamental geomorphic factors of the small watershed are analyzed, including the watershed area (F), watershed shape coefficient (Ke), average slope (S), gully density (Ds), hypsometric integral (HI), and gully gradient (J). Moreover, an attempt is made to identify two key factors governing debris flow hazards: (1) the scale of debris flows and (2) the influence of regional tectonic stress on debris flow gullies.

In the quantitative assessment of the first key factor—debris flow scale, a two-pronged approach is adopted. Firstly, a comprehensive analysis is carried out on the unique characteristics of debris flow material sources derived from field investigations. Geographic Information System (GIS) technology and remote sensing images are utilized for the classification and detection of material sources, and the volumes of solid material (V_s_) in different stratigraphic lithologies are quantified. Secondly, the rainstorm-flood method is employed to calculate the total volume of debris flow discharge per event (Q_t_). Through regression analysis, mathematical relationships are established among Q_t_, Vs, and geomorphic indices (gully gradient (J), watershed area (F), and relative elevation (H)), followed by the development of predictive models for hazard range (width B and length L).

**Fig 6:**
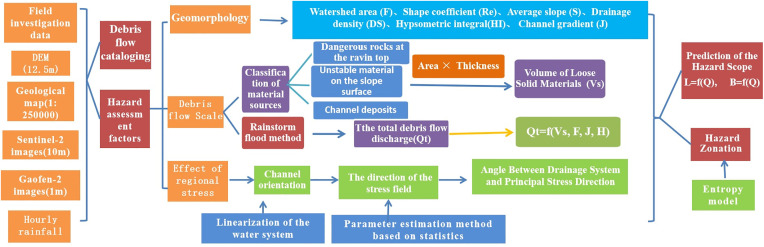
Flowchart of debris flow hazard assessment.

For the second key factor, considering the distribution pattern of the water system under tectonic control, a quantitative method is proposed. Finally, by integrating three sets of quantitative factors-geomorphic parameters, the volume of debris flow solid material, and the angle between the water system direction and the principal stress direction-and applying the principle of disaster entropy, a comprehensive model for evaluating the hazards of clustered debris flows in small watersheds is established. The detailed flowchart is illustrated in Fig 6.

### 3.1. Selection and processing of influencing factors

#### 3.1.1. Geomorphic factors.

In the hazard analysis of clustered debris flows, six essential geomorphic factors have been selected. All of these can be derived and calculated from ALOS-DEM source data via the ArcGIS platform.

(1) Watershed area (F)

The watershed area refers to the catchment range enclosed by the watershed divide. It serves as a vital parameter for evaluating the hydrodynamic conditions of a gully. Generally, a larger watershed area is more conducive to favorable hydrodynamic conditions. In the context of debris flow activities, the watershed area of a gully is most beneficial when it lies within a particular range.

(2) Watershed shape coefficient (Ke)

The watershed shape coefficient (Ke) is defined as the ratio of the actual length (L) of the watershed divide to the circumference (LA) of a circle having the same area as the watershed. The coefficient reflects the isochrones of rainfall and the occurrence of peak confluence flood values, thereby exerting an influence on erosion and sedimentation within the watershed. A watershed shape coefficient value closer to 1 indicates a more circular watershed shape, faster confluence speed, and a higher likelihood of large floods forming, which may subsequently cause debris flows.

(3) Average Slope (S)

The slope influences surface runoff. The steeper the slope, the more rapidly the watershed runoff reaches its peak, and the higher the peak flow. The surface of the slope is more susceptible to erosion and scouring, forming sources of slope material. Simultaneously, with the increase in slope steepness, the potential energy of loose materials rises, and their response to environmental changes becomes more significant, which promotes the formation and movement of debris flows. Field investigations have indicated that the majority of material sources in the study area are distributed within the range of 10° to 40°.

(4) Gully Density (D_s_)

Gully density (D_s_) is defined as the ratio of the total length of gullies (∑L) within a gully system to the total area (F). It indicates the extent of land surface fragmentation caused by water flow and can comprehensively mirror the impact of factors such as climate, terrain, lithology, and vegetation in the study area on the development and activity of debris flows. The higher the gully density implies a more severe incision of the land surface by water flow and a greater fragmentation of surface gullies. This heightens the probability of the formation of solid-liquid two-phase runoff and elevates the likelihood of debris flow occurrence.

(5) Hypsometric Integral (HI)

Research findings have demonstrated that the hypsometric integral (HI) index as an indicator of the erosion degree within a watershed. A relatively higher HI value signifies a younger evolutionary phase of the watershed and enhanced erodibility [14]. By employing the hypsometric integral, it enables the classification of the geomorphic development stages of debris flow gullies, which quantitatively reflects the region’s capacity to supply loose materials.

The hypsometric integral (HI) is calculated using the formula:


$$\begin{array}{c} \text{HI }=({\text{H}}_{\text{mean}}-{\text{H}}_{\text{min}})/({\text{H}}_{\text{max}}-{\text{H}}_{\text{min}}), \end{array}$$

where, H_mean_, H_max_, and H_min_ represent the average, maximum, and minimum elevations within the catchment, respectively.

(6) Gully Gradient (J)

The longitudinal gradient of the main gully significantly influences the rate at which precipitation converges into the gully over the same time period. This convergence leads to an augmentation of water flow’s velocity and kinetic energy, consequently enhancing its ability to transport loose materials within the gully. This phenomenon provides favorable hydrodynamic conditions for the formation of debris flows. Moreover, a steeper longitudinal gradient contributes to higher velocities and stronger kinetic energy for debris flows during their movement, resulting in increased destructiveness and extended propagation distances.

#### 3.1.2. Key factors of debris flow hazard.

Debris Flow Scale (Q_t_) Derives From Source Characteristics. Loose solid materials serve as the basis of debris flow activities. Their distribution, reserves, and geotechnical characteristics influence the probability of debris flow occurrence and the extent of potential damage, making them a critical factor in assessing debris flow hazards. Conversely, there are generally two approaches to calculating the scale of debris flows. One method employs theoretical expressions that consider the duration of a debris flow event and its flow rate [15, 16], while the other derives empirical relationships between the scale of a debris flow and one or more factors from historical debris flow data [17]. Research findings [18–21] suggest that the total debris flow discharge volume per event is closely linked to the watershed area and the total quantity of loose solid materials within the watershed. Consequently, based on the total debris flow discharge volume per event in 24 debris flow gullies in the Tieba River Basin under the condition of a rainfall frequency of once every 200 years, a prediction model for the scale of debris flow was constructed using the regression analysis method. This model quickly and quantitatively expresses the volume of debris flow discharge per event in the study area with fewer factors, which facilitates hazard assessment and disaster prevention and mitigation.

(1) Volume of Loose Solid Materials (V_s_)

Due to variations in their formation causes, topographic and geomorphic characteristics, the thickness of deposits, duration, and degree of consolidation, the stability of solid material sources in debris flow gullies can differ significantly, as can the likelihood of them becoming debris flow material sources. Field investigations revealed multiple debris flow gullies on both banks of the Tieba River Basin. There are essentially no newly formed landslides within the basin. However, a substantial amount of debris accumulates at the bottoms of the main debris flow gullies and their tributaries, as well as on the slopes flanking the gully channels. These accumulations primarily consist of unconsolidated and weakly consolidated collapsed rocks. Consequently, by analyzing remote sensing data and conducting on-site inspections (a total of 96 measurement points that are evenly distributed in 24 gullies have been measured by laser rangefinder and trench measurement), the material sources in the Tieba River Basin are predominantly categorized into three major groups.

**Fig 7:**
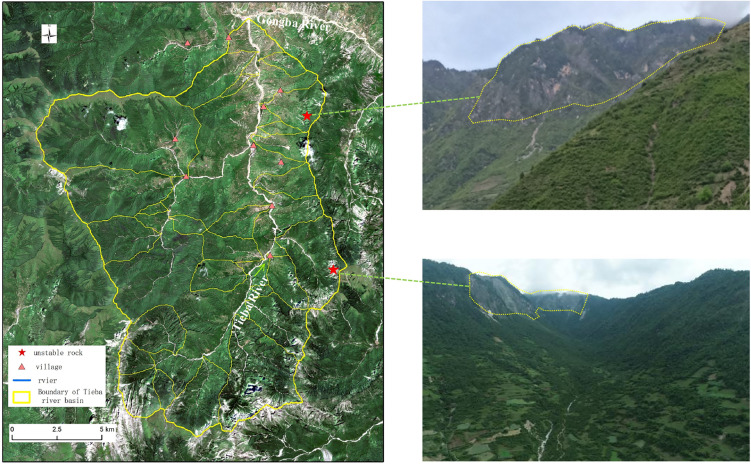
Unstable rocks at the rear edge of the debris flow formation area (The Sentinel-2 imagery is publicly available from the Copernicus Open Access Hub (https://scihub.copernicus.eu). The rocks consist of medium-thick-layered limestone from the Triassic system, with an average thickness of 25 m).

The first group consists of unstable rocks at the summit, primarily referring to potential collapse bodies formed by the thick-layered limestone on the rear watershed of the formation area within the basin, influenced by structural joints and weathering (Fig 7). These rocks significantly contribute to the loose accumulations in the debris flow gullies. The lithology of the strata in this region predominantly consists of medium-thick-layered limestone from the Triassic system, with a weathered layer thickness ranging from 18 to 25 meters. For the estimation of material source reserves in this case, an average thickness of 25 meters is utilized.

**Fig 8:**
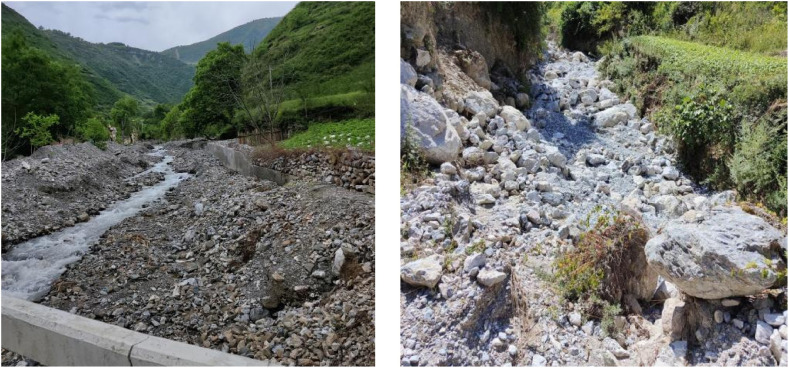
Deposits in the debris flow gully channel (the channel sediment thickness is estimated to be 2 m).

The second group pertains to channel deposits. The excavation and exposure of gully channel deposits are crucial for assessing debris flow reserves. Field investigations of recent debris flows in the Tieba River Basin indicate that gully beds have been filled with sediment to a depth of 2–7 meters. Research findings as documented in the “Code for Exploration of Engineering for Prevention and Control of Debris Flow Disasters (T/CAGHP 006-2018) [22]” reveal that a single rainfall event can cause scouring and exposure of debris flow deposits to a depth ranging from 0.2 to 2 m. Given the short time since the disaster event occurred, the consolidation of the gully channel deposits is minimal (Fig 8). Therefore, for the material source reserve calculations in this study, an estimated uncovering thickness of 2 meters has been used.

**Fig 9:**
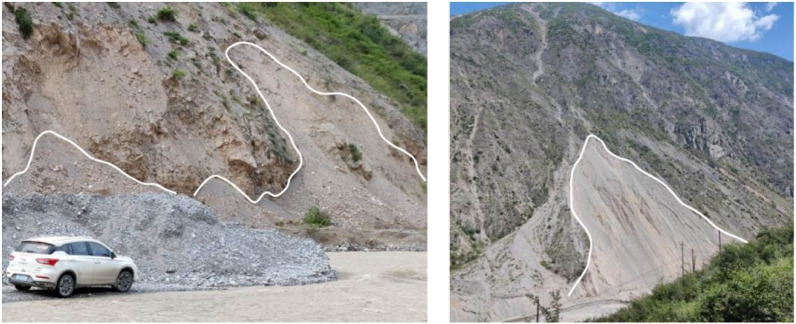
Unstable material sources of loose colluvial deposits on the slope surface and the talus cone at the toe of the slope (The thickness of colluvial deposits on the slope surface is estimated to be 1 m).

The third group encompasses unstable material sources on the slope surface, primarily consisting of newly formed colluvial deposits (Fig 9). These deposits, typically situated on the concave bank, are eroded by water flow within the gully. Distributed along the hillside, they resemble an obtuse triangle, with a thinner upper portion and a thicker base. Following heavy rainfall, these loose accumulations can be readily transported and swept away by the water flow. The average thickness of the loose materials that can be mobilized on the slope surface of the debris flow gully is approximately 1 meter.

The estimation of material sources is typically calculated by multiplying the distribution area by the average thickness [22], after establishing both the distribution area and the average thickness. This is represented by Equation (1).


$$\begin{array}{c} V_{\text{s}}=A\overset{―}{h} \end{array}$$ (1)

Where, *V*_*s*_ is the volume of the solid material (m^3^);$\overset{―}{h}$ is the average thickness of the solid material (m); A is the distribution area of the solid material (m^2^).

(2) The total volume of debris flow discharge per event (Qt)

Two common methods for calculating debris flow discharge are the morphological investigation method and the rainstorm flood method. Studies show that the former yields slightly lower values than the latter [23], as it only reflects a single event, while the rainstorm flood method incorporates watershed area, rainfall frequency, runoff coefficient, and vegetation coverage, offering greater accuracy. Thus, the latter is adopted here.

The rainstorm flood method assumes debris flow timing/frequency matches rainfall events, with no water loss to evaporation or infiltration, converting all floodwater into debris flow discharge. The steps include: Calculating design discharge for rainstorm floods at different frequencies; Setting a blockage coefficient based on gully features; Deriving peak debris flow discharge; and Estimating total debris volume per event under the design rainfall frequency.

① Calculation of the water flow peak discharge of debris flow

Since there is no measured discharge data, the peak discharge of water flow in the debris flow (Q_b_) can be calculated according to the formula provided in the hydrological manual [24]. The calculation formula is as follows:


$$\begin{array}{c} {\text{Q}}_{\text{b}}\text{= 0.278}\psi \frac{\text{S}}{\tau^{\text{n}}}\text{F} \end{array}$$ （2）

where Q_b_ is the peak discharge of water flow (m^3^/s); ψ is the peak runoff coefficient; S is the rainfall intensity of the rainstorm, that is, the maximum rainfall amount in 1 hour (mm/h); τ is the watershed concentration time, in hours; n is the rainstorm parameter; F is the catchment area of the small watershed (km^2^).

② Peak discharge of debris flow (Q_c_)

Since 1959, building upon the verification of debris flow discharge calculation formulas proposed by various researchers, the debris flow discharge formula has been refined as follows [25]:


$$\begin{array}{c} Q_{c}=(\text{1}+\varphi )Q_{b}D_{c} \end{array}$$ （3）

where Qc is the peak discharge of debris flow (m^3^/s) with a frequency of P; φ is the sediment correction coefficient of debris flow, which is calculated according to Equation (4); Qb is the design discharge of rainstorm flood (m^3^/s) with a frequency of P; Dc is the blockage coefficient of debris flow.


$$\begin{array}{c} \varphi =\frac{\gamma_{c}-\gamma_{w}}{\gamma_{s}-\gamma_{c}} \end{array}$$ （4）

where γ_c_ is the unit weight of the debris flow (t/m^3^); γ_w_ is the unit weight of water flow (t/m^3^); γ_s_ is the unit weight of solid matter (t/m^3^).

Previously, the in-situ slurry preparation method was commonly employed to determine the unit weight γc of debris flows. This method necessitates on-site proportioning and verification of the fluid, which is highly susceptible to human influence and subjective judgment. To objectively assess the unit weight of debris flows, experts and scholars have conducted extensive research and obtained numerous results [25–27]. Notably, the formula that combines the content of coarse particles (grain size > 2 mm) and clay particles (grain size < 0.05 mm) [28] exhibits superior universality. Consequently, this paper utilizes Equation (5) [28], based on laboratory particle size analysis tests of 14 debris flow samples collected from the field, to calculate the unit weight of debris flows in the Tieba River Basin.


$$\begin{array}{c} \gamma_{c}=P_{\text{0}\text{5}}^{\text{0}.\text{3}\text{5}}P_{\text{2}}\gamma_{v}+\gamma_{\text{0}} \end{array}$$ （5）

where γ_c_ is the unit weight of the debris flow (t/m^3^); P05 is the percentage of fine particles with a size smaller than 0.05 mm (represented by a decimal); P2 is the percentage of coarse particles with a size greater than 2 mm (represented as a decimal); γ_v_ is the minimum unit weight of viscous debris flow (2.0 t/m^3^); γ_0_ is the minimum unit weight of debris flow (1.5 t/m^3^).

③ Total Volume of Debris Flow Discharge per Event (Q_t_)

The calculation methods for the total volume of debris flow discharge during a single debris flow event can be categorized into two approaches. First, for debris flows where all solid materials settle on the accumulation fan following the event, the total volume of solids can be determined by measuring the length, width, and thickness of the deposited materials. Second, the approach involves predicting and calculating the total volume of debris discharged in a single event. The methods for prediction and calculation are twofold: (1) Empirical formula methods for continuous debris flows, which include empirical models based on the statistical relationship between the total volume of solids and the peak discharge of debris flows, the pentagon method, and the modified pentagon method; (2) For episodic and mixed debris flows, the discharge volume and movement duration are counted, and a statistical model is established by considering the relationship between flow interruption time, the discharge of the debris flow’s leading part, and the total volume. Given that this paper focuses on the prediction and evaluation of hazards and recognizing the significant human activity at the mouths and within the debris flow gullies in the Tieba River Basin, where local residents actively engage in disaster relief efforts, most of the deposits on the debris flow accumulation fans have been mechanically cleared. Consequently, the conditions for measuring the total volume in a single debris flow event are no longer met. Therefore, based on the duration of the debris flow and the maximum discharge, and considering the abrupt rise and fall characteristics of the debris flow, the process line is generalized into a pentagon shape, and the calculation is performed using Equation (6) [26].


$$\begin{array}{c} Q_{t}=0.264TQ_{c} \end{array}$$ （6）

where Q_t_ represents the total volume of debris flow discharge per event (m^3^); T is the duration of the debris flow (s); and Qc is the peak discharge of the debris flow (m^3^/s).

(3) Relationship for V_s_ and Q_t_

To ascertain the correlation between the volume of debris flow discharged in a single event (Q_t_) in the Tieba River Basin and the watershed area (F), the volume of loose solid materials (V_s_), the relative elevation difference of the watershed (H), and the total gradient of the main gully (J), regression analysis was performed on the data from 24 debris flow gullies within the study area. Both Q_t_ and Vs can be computed using the methods outlined in the preceding sections, while the geomorphic parameters F, J, and H can be derived and extracted via DEM and GIS. Following the regression analysis, for gullies under comparable conditions, Q_t_ can be determined using the subsequent formula.


$$\begin{array}{c} Q_{t}=f\left(V_{s},F,J,H\right) \end{array}$$ (7)

where Q_t_ is the total volume of debris flow discharge per event (10^4^ m^3^); V_s_ is the volume of the solid material within the single watershed (10^4^ m^3^); F is the area of the watershed (km^2^); J is the longitudinal gradient of the main gully; H is the elevation difference of the watershed (km).

**3.1.2.2 The Tectonic Impact.** Tectonic conditions play a fundamental role in controlling debris flow initiation, material supply, and channel development. The regional river system reflects the interplay between tectonic uplift and erosion. External forces (e.g., rainfall, weathering) introduce randomness in drainage directions, while tectonic forces impose systematic patterns [28, 29]. Steep gradients caused by tectonic uplift promote rapid runoff, enhancing debris flow transport capacity [30].

The Tectonic angle (T), which is the angle of intersection between the main gully’s direction and the stress field’s orientation, is selected as the factor to quantify the influence of regional tectonic stress on the water system pattern [31–34]. It is defined as the angle between the average direction of the primary debris flow gully and the direction of the principal compressive stress of the tectonic stress field. When the principal compressive stress’s direction is nearly perpendicular to the average direction of the debris flow gully, the stability of the gully walls in such a debris flow gully is compromised, making landslides likely to occur. Erosion is exacerbated, and the debris flow can acquire a substantial supply of material. Therefore, this angle (T) is selected as one of the key factors to quantify the hazard of debris flow in this analysis.

The dominant direction of the river system can be intuitively determined through the direction rose diagram. Generally, there is a relatively large error. It is usually easy to determine the first dominant direction, but the second dominant direction is difficult to define. The main methods for determining the dominant direction through data calculation and analysis are as follows: Kohlbeck and Scheidegger (1977) [29] proposed a parameter estimation method based on statistics, and YU (1985) [28] proposed a simplified algorithm for density. The former requires the given probability density function, and it is not easy to specify the probability density function due to various limitations. Therefore, in this paper, the simplified algorithm for density proposed by YU (1985) [28] is used to calculate the tectonic state reflected by the river system pattern in the Tieba River Basin.

The calculation process is as follows: Divide the directions of the river system into groups with an interval of 12°. A total of 180° is divided into 15 groups, and then rearranged according to the magnitude of the angles to obtain [(θ_0_,l_0_),(θ_1_,l_1_),(θ_2_,l_2_),…,(θ_15_,l_15_)]. Sum up the lengths of the river systems within each group and multiply them by a weight sequence of the normal distribution to obtain the density${\overset{―}{L}}_{\text{n}}$.


$$\begin{array}{c} {\overset{―}{L}}_{n}=\sum_{i=-k}^{k}\lambda_{i}L_{n+i}(n=\text{1},\text{2},\text{3},,\text{1}\text{5}) \end{array}$$ (8)

where ${\overset{―}{L}}_{\text{n}}$ is the density of the distribution direction of the river system at θn with respect to the weight Λ;

Λ =(l_-k,_l_-k+1,_…l_1,_l_0,_l_1,_…l_k-1,_l_k) is_ a selected weight sequence of the normal distribution; Ln is the sum of the lengths of the river systems within the group.

Λ =(l_-k,_l_-k+1,_…l_1,_l_0,_l_1,_…l_k-1,_l_k_)=[c, c + b, c + 2b,…,c+(k-1) b,c + kb,c+(k-1) b,…c + 2b, c + b,c] is a set of non-negative numbers, symmetric numbers, with _0_ being the maximum value, and it strictly decreases towards both ends in sequence. For the calculation, we can set c = 2^n,^ b = 1, and call Λ a set of weights.

### 3.2. Evaluating the hazard of debris flows in small mountainous watersheds

#### 3.2.1. Prediction of the hazard scope of debris flows.

Based on the remote sensing image data of the study area, in conjunction with field investigation and measurement results, it has been statistically determined that there are 11 debris flows with intact accumulation fans. By integrating high-resolution remote sensing images with a 1-meter resolution and employing ArcGIS, the parameters of these 11 debris flow channels can be derived. Regression analysis should be conducted using the measured data to establish formulas for estimating the length (L) and width (B) of debris flow deposits using the total volume of debris flow discharged in a single event (Qt) as a variable. Consequently, the calculation formula for the debris flow hazard area has been obtained, allowing for the prediction of the hazard scope of debris flows in other gullies. The deposit’s length and width are computed using these formulas, and the shape of the newly formed debris flow accumulation fan is determined by comparing it with high-definition images.


$$\begin{array}{c} B=f\left(Q_{t}\right);L=f(Q_{t}) \end{array}$$

This section analyzes hazard zoning for debris flows in small mountainous watersheds, incorporating the information entropy model. Entropy measures the uncertainty within a system following the influence of various interacting factors. To evaluate the m debris flow gullies, the occurrence of debris flow disasters in each watershed is collectively impacted by n influencing factors. Construct a mathematical model of “disaster entropy” using the following steps [35, 36], and employ this model to ascertain the hazard of each debris flow gully.

#### 3.2.2. Evaluation of regional debris flow hazard.

When evaluating the hazard of regional debris flows using disaster entropy, it is essential to begin by considering the disaster-causing background of the area to select evaluation factors that significantly influence the occurrence of debris flows. Subsequently, calculate the value of each evaluation index based on the evaluation units and construct a debris flow disaster evaluation matrix. However, since the attributes of each evaluation factor are not identical, it is necessary to standardize each factor and convert them into dimensionless data for direct comparison. The hazard value (Pi) of each evaluation unit should be calculated using Formulas 9–13.


$$\begin{array}{c} F_{\text{ij}}=r_{ij}/\sum_{j=1}^{n}r_{ij} \end{array}$$ (9)

$$\begin{array}{c} E_{j}=-K\sum_{i=1}^{m}F_{ij}lnF_{ij} \end{array}$$ (10)

$$\begin{array}{c} V_{\text{j}}=1-E_{j} \end{array}$$ (11)

$$\begin{array}{c} w_{j}=V_{j}/\sum_{j=1}^{n}V_{j} \end{array}$$ (12)

$$\begin{array}{c} P_{i}=\sum_{j=1}^{n}w_{j}r_{ij} \end{array}$$ (13)

Where r_ij_ is the standardized value of the j-th evaluation index of the i-th evaluation unit; F_ij_ is the frequency of the occurrence of the evaluation index j in the process of debris flow disaster generation; E_j_ is the disaster entropy of the j-th evaluation index; K = 1/lnm; m is the number of evaluation units; V_j_ is the index utility value of the j-th evaluation index; w_j_ is the weight of the j-th evaluation index; Pi is the hazard value of debris flow disaster in the i-th evaluation unit.

### Field study permits

No specific permits were required for the described field studies. The study locations are not privately owned or protected, and the field studies did not involve endangered or protected species.

## 4. Results

### 4.1. Calculation results of key parameters

#### 4.1.1. Scale of debris flow.

**Fig 10:**
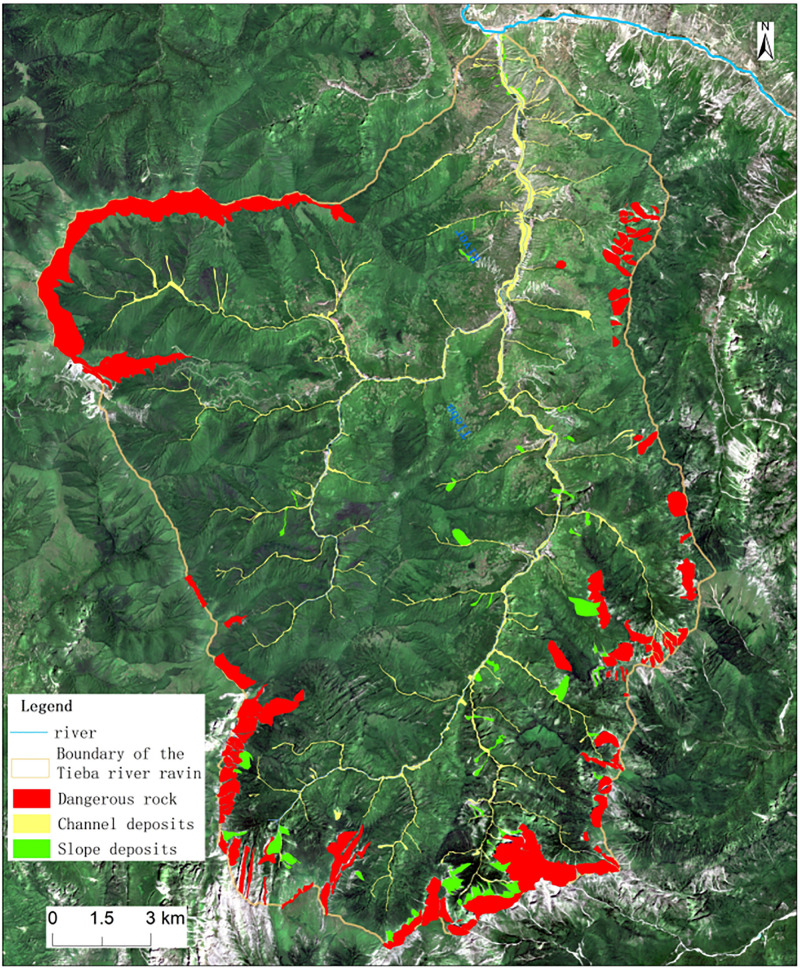
Spatial distribution of three types of loose solid material sources in the Tieba River Basin (interpreted from 1 m resolution high-resolution remote sensing images; material sources include unstable rocks, gully deposits, and slope colluvium; Sentinel-2 data © Copernicus Open Access Hub 2020).

(1) Volume of Solid Material (Vs) Utilizing field investigation data, this paper employs the ARCGIS platform to conduct the interpretation of high-definition remote sensing images (at a 1-meter resolution) in the Tieba River Basin using a method of human-computer interactive visual interpretation. The distribution locations, areas, and shapes of the material sources in the 24 debris flow watersheds of the Tieba River have been identified (refer to Fig 10). Comparison results of pre- and post-disaster high-resolution remote sensing images (10 m Sentinel-2 and 1 m domestic high-resolution images) show no large-scale landslide scars. Field survey records of 24 debris flow gullies confirm no large-scale landslide supply. Lithology and structural characteristics of the basin are not conducive to the formation of large-scale landslides. By analyzing the characteristics of each type of material source and incorporating the average thickness estimated from field investigations, the reserves of material sources in each debris flow gully within the Tieba River Basin can be calculated using Formulas (5–12). The results of these calculations are presented in Table 1.

**Table 1:** Reserves of loose materials (V_S_) in debris flow gullies of the Tieba river basin.

Debris Flow Number	Gully Deposits (m^3^)	Unstable Rocks (m^3^)	Slope Deposits (m^3^)	Total Amount of Material Sources (10^4^ m^3^)
1	164896	0	0	16
2	143975	677038	0	82
3	38732	18873554	0	1891
4	52869	0	0	5
5	1101291	107244558	0	10835
6	202608	17716023	0	1792
7	124904	0	12311	14
8	12632	0	0	1
9	79938	0	65928	15
10	41788	0	0	4.
11	118940	0	34862	15.
12	81279	0	113650	19
13	16629	16392849	259405	1667
14	92610	0	28947	12
15	39059	0	0	4
16	94556	22263342	0	2236
17	222245	14654022	198175	1507
18	635594	74519821	714261	7587
19	55482	16827574	112502	1700
20	11259	4274892	0	429
21	215174	20838087	96002	2115
22	286760	27883821	30902	2820
23	229421	7179619	0	741
24	5887	1057203	0	106

As indicated in Table 1, within the Tieba River Basin, among the 24 debris flow gullies, there are 407 × 10⁴ m^3^ of gully deposits, 2803 × 10⁴ m^3^ of potential unstable rocks, 167 × 10⁴ m^3^ of slope material sources, and the total reserve of loose materials amounts to 3377 × 10⁴ m^3^.

(2) The total volume of debris flow discharge per event (Qt)

The soil samples collected from the debris flow accumulation zone were subjected to particle size distribution tests, and the results are presented in Table 2. Based on these experimental results, the unit weight of the debris flow (γc) was determined as the average of 16 samples, resulting in γ_c_ = 1.875 t/m^3^. Meanwhile, the unit weight of water flow was taken as γw = 1 t/m^3^. Field observations showed that the debris flow deposits in the study area mainly consist of limestone, leading to the assumption that the solid material unit weight is γ_s_ = 2.70 t/m^3^.

**Table 2:** Particle size distribution data and unit weight calculation of debris flow.

Sample No.	Particle Composition %											Unit Weight (t/m^3)^
	Gravel					Sand			Silt		Clay	
	Coarse			Medium	Fine	Coarse	Medium	Fine				
Particle Size (mm)	＜80	＜60	＜20	＜10	＜5	＜2	＜0.5	＜0.25	＜0.05	＜0.01	＜0.005	
1	100	97.56	80.56	53.53	35.63	18.69	8.95	3.66	1.53	0.56	0.21	1.834
2	100	99.2	77.7	54.4	34.2	16.2	6.4	4.82	0.97	0.27	0.15	1.834
3	100	98.9	64.4	42.9	29	21.9	16.03	11.71	1.31	0.56	0.34	1.842
4	100	96.53	69.62	38.56	28.75	20.86	15.96	10.89	1.36	0.69	0.46	1.842
5	100	87.8	54.5	39.6	29.7	22.8	16.23	10.44	1.88	0.75	0.51	1.922
6	100	98.3	68.6	45.9	27.5	16.6	9.57	6.4	0.13	0.06	0.04	1.649
7	100	84.4	51.2	39.7	31.8	25.2	16.43	10.63	2.03	0.68	0.43	1.906
8	100	94.4	72.2	55	41.5	31.8	22.64	14.97	2.06	0.82	0.6	1.895
9	100	91.9	62	44.1	31.4	24	17.86	12.11	2.17	0.74	0.52	1.943
10	100	96.9	81.1	63.2	48.1	36.9	26.32	19.35	4.13	1.41	0.98	1.988
11	100	100	64.8	48	35.1	26.9	19.4	13.14	1.91	0.64	0.45	1.896
12	100	91.2	59.7	45.2	36	28.7	20.09	13.67	4.02	1.98	1.42	2.014
13	100	100	82.1	70.5	59.9	50.2	35.85	24.03	1.59	0.61	0.4	1.734
14	100	92.8	58.1	42.9	33.8	27.3	19.59	13.6	2.81	1.33	0.99	1.956
15	100	97.4	57.5	35.9	22.6	15.2	9.92	7	1.24	0.36	0.25	1.878
16	100	97	66.6	54.1	45.9	39.7	32.34	21.65	0.39	0.07	0.03	1.674

According to the “Comprehensive Plan for Post-Disaster Recovery and Reconstruction of Rainstorm and Flood Disasters in Longnan and Other Areas issued by Gansu Province,” the rainfall in August 2020 in the Tieba river basin (Qugao’na section of the Gongba river) reached a design recurrence interval of once in 200 years. Consequently, the design rainstorm frequency was calculated as P = 0.5%. Utilizing this frequency (P = 0.5%), the rainfall values were derived from the rainfall isohyet map, along with the coefficient of variation.

Field investigations of the debris flow gullies in the Tieba river basin revealed the following characteristics: (1) The gullies exhibit moderate curvature, lacking sharp bends; (2) The channel width varies significantly; (3) Local steep steps are present, and abundant loose sediment deposits cause partial blockage of the channels. Consequently, a blockage coefficient of Dc = 2.5 was adopted. Utilizing the above parameters and Equation 2–6, calculations were performed for the 24 debris flow gullies in the Tieba river basin under the 1-in-200-year rainstorm scenario. The results include the clear-water peak discharge (Q_b_), debris flow peak discharge (Q_c_), and the total volume of a single debris flow event (Q_t_). Thus, under a design rainstorm scenario with a 200-year recurrence interval, the total volume of a single debris flow event across the 24 gullies in the Tieba river basin is calculated to be 17.8534 million cubic meters.

(3) Regression analysis between debris flow scale (Q_t_) and the other four parameters

**Fig 11:**
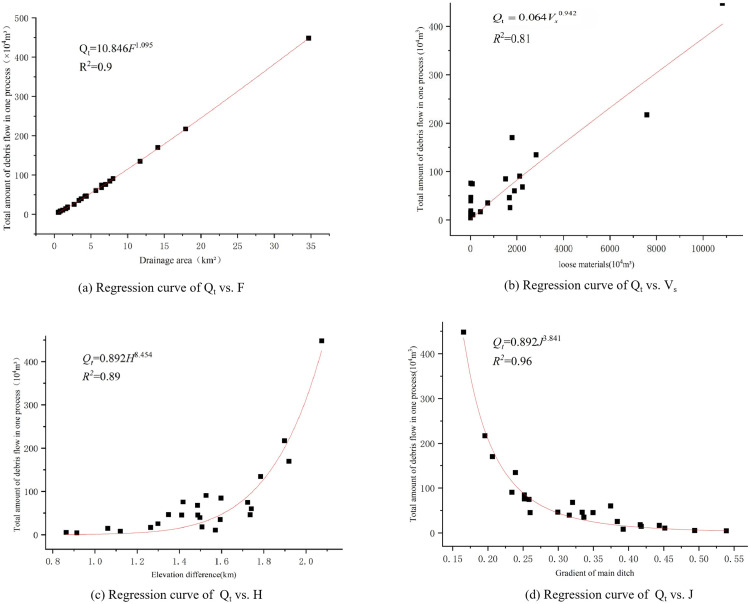
Regression curves (the specific parameters of each debris flow channel are available in the dataset (https://osf.io/3kgjd/overview?view_only=a20df6c94339414bb7b62780ca618ec1)).


Regression analysis was conducted on the total volume of debris flow discharge per event (Q_t_) for debris flows in 24 gullies within the study area (Fig 11), considering factors such as watershed area (F), source material volume (V_S_), relative watershed elevation difference (H), and the main gully total gradient (J). The following formulas were derived.

$$\begin{array}{c} Q_{\text{t}}=10.846F^{1.095} \end{array}$$ (14)

$$\begin{array}{c} Q_{\text{t}}=0.0641{V_{s}}^{0.942} \end{array}$$ (15)

$$\begin{array}{c} Q_{t}=0.892H^{8.454} \end{array}$$ (16)

$$\begin{array}{c} Q_{t}=0.892J^{-3.841} \end{array}$$ (17)

The test results indicated that the R^2^ values for each set of data were 0.9, 0.81, 0.81, and 0.96, respectively, signifying a good regression effect (Fig. 11).

In summary, a nonlinear functional relationship has been established between the total volume of debris flow discharge per event (Qt) and four parameters (Vs, F, J, H) for the 24 debris flow gullies in the study area. This relationship serves as a predictive model for debris flow scale, with the mathematical expression as follows:


$$\begin{array}{c} Q_{t}=0.077(V_{s}/F{)}^{0.818}+0.075J^{-4.725}+4.93H^{3.385} \end{array}$$ (18)

The multiple correlation coefficient (R^2^ = 0.87) of the debris flow scale prediction model meets the correlation test requirements, demonstrating a statistically significant nonlinear regression effect. Error analysis confirms that the precision remains within an acceptable range.

**Fig 12:**
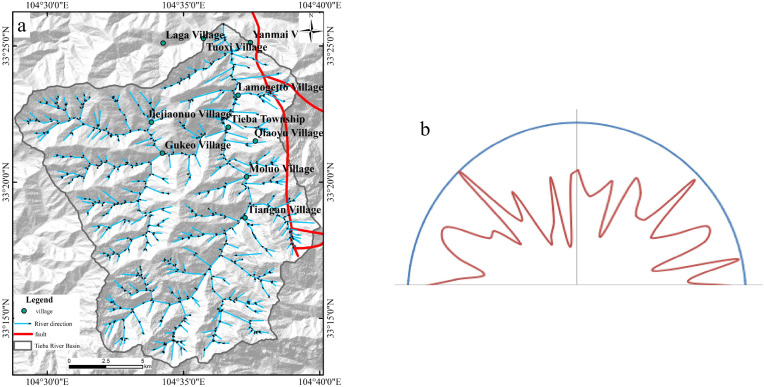
Calculation of drainage orientation (the specific parameters of each debris flow channel are available in the dataset (https://osf.io/3kgjd/overview?view_only=a20df6c94339414bb7b62780ca618ec1), Shaded relief map of the study area derived from ALOS 12.5 m DEM data were obtained from the Geospatial Data Cloud (http://www.gscloud.cn). Figure (http://www.gscloud.cn). Figure(a) shows drainage distribution map of the Tieba River basin; Figure (b) is the rose diagram statistics of channel orientations).

**4.1.2 Angle Between the River System and the Principal Stress Direction (T). Using** ALOS-DEM data with a resolution of 12.5 meters, a threshold value of 800 was selected in ArcGIS software to extract the river system across the entire study area. The azimuth angle of the average direction of each river system was calculated using the spatial statistics tool in ArcGIS, and a rose diagram of the river system was created with a grouping interval of 5 degrees for the results (Fig 12).

Based on the river network distribution within the study area, three predominant orientations were determined: Max1 = 6°, Max2 = 138°, and Max3 = 90°. By drawing an analogy between the entire study area and a rock-soil mass, the principal compressive stress direction of the tectonic stress field can be compared to the axial pressure in a triaxial shear strength test of the rock-soil mass. The two shear fracture zones generated by the tectonic stress field are analogous to the two shear failure planes formed during rock-soil mass failure.

According to rock-soil mechanics, in a triaxial shear strength test, the axial pressure direction typically aligns with the bisector of the acute angle between the two shear planes of the rock-soil mass. Furthermore, as the confining pressure increases, the corresponding acute angle decreases. Consequently, if the two primary orientations of the river system are interpreted as a set of shear zones developed under the tectonic stress field, then the bisector of their acute angle indicates the principal compressive stress direction of the tectonic stress field.

The bisector of the acute angle between Max1 (6°) and Max2 (138°) is 66°, indicating a principal stress field orientation of NW66°. This direction is nearly parallel to the strike of the major fault in the region and approximately perpendicular to the strike of secondary faults. Furthermore, it aligns precisely with the dip direction of the rock layers in the rear collapse zone, significantly exacerbating slope instability.

The bisector of the acute angle between Max1 (6°) and Max3 (90°) is 48°, corresponding to a principal stress field orientation of NE48°. This direction forms an angle of approximately 70° with the major fault (a reverse fault) in the area, consistent with previous studies [37, 38].

### 4.2. Debris flow hazard assessment

#### 4.2.1. Prediction of hazard zones for debris flow gully.

Based on remote sensing imagery of the study area and field survey measurements, 11 debris flow gullies with complete alluvial fans were identified. Utilizing high-resolution remote sensing images (with a 1-meter resolution and ArcGIS, the parameters of these 11 debris flow gullies were obtained (Table 3).

**Table 3:** Geometric parameters of debris flow deposition fans.

Debris Flow Watershed No.	Q_t_(10^4^m^3^)	Length of Debris Fan (km)	Width of Debris Fan (km)
2	74.67	0.167	0.238
4	8.29	0.118	0.145
5	447.97	0.413	0.308
12	46.35	0.131	0.165
13	46.01	0.084	0.14
17	84.71	0.14	0.39
18	217.28	0.194	0.48
20	16.75	0.133	0.109
21	90.57	0.161	0.152
24	10.73	0.112	0.141

Regarding the relationship between maximum deposition length, maximum deposition width, and debris flow volume, numerous scholars have derived relevant findings through experiments and numerical simulations [17,39–41]. For instance, Rickenmann [17] established an empirical debris flow model in an exponential form, correlating the maximum runout length in deposition zones with total debris flow volume and elevation loss along the flow path. In this study, based on fundamental data from 11 debris flow gullies in the research area, regression analysis was conducted to develop calculation formulas for predicting maximum deposition length and width, using debris flow discharge volume as the key parameter.


$$\begin{array}{c} L=\text{0}.\text{0}\text{2}\text{4}{Q_{t}}^{\text{0}.\text{4}\text{4}\text{1}} \end{array}$$ (19)

$$\begin{array}{c} B=\text{0}.\text{0}\text{6}\text{6}{Q_{t}}^{\text{0}.\text{2}\text{9}\text{1}} \end{array}$$ (20)

In the equations: Q_t_ represents the one-time debris flow discharge volume, L denotes the maximum deposition length, with a multiple correlation coefficient R^2^ = 0.76, indicating statistically significant regression. B stands for the maximum deposition width, showing a correlation coefficient R^2^ = 0.49. Error analysis through self-verification using deposition fan data from the 11 debris flow gullies confirmed that all precision indicators remain within acceptable control limits.

#### 4.2.2. Regional Debris Flow Hazard Zoning.

**Fig 13:**
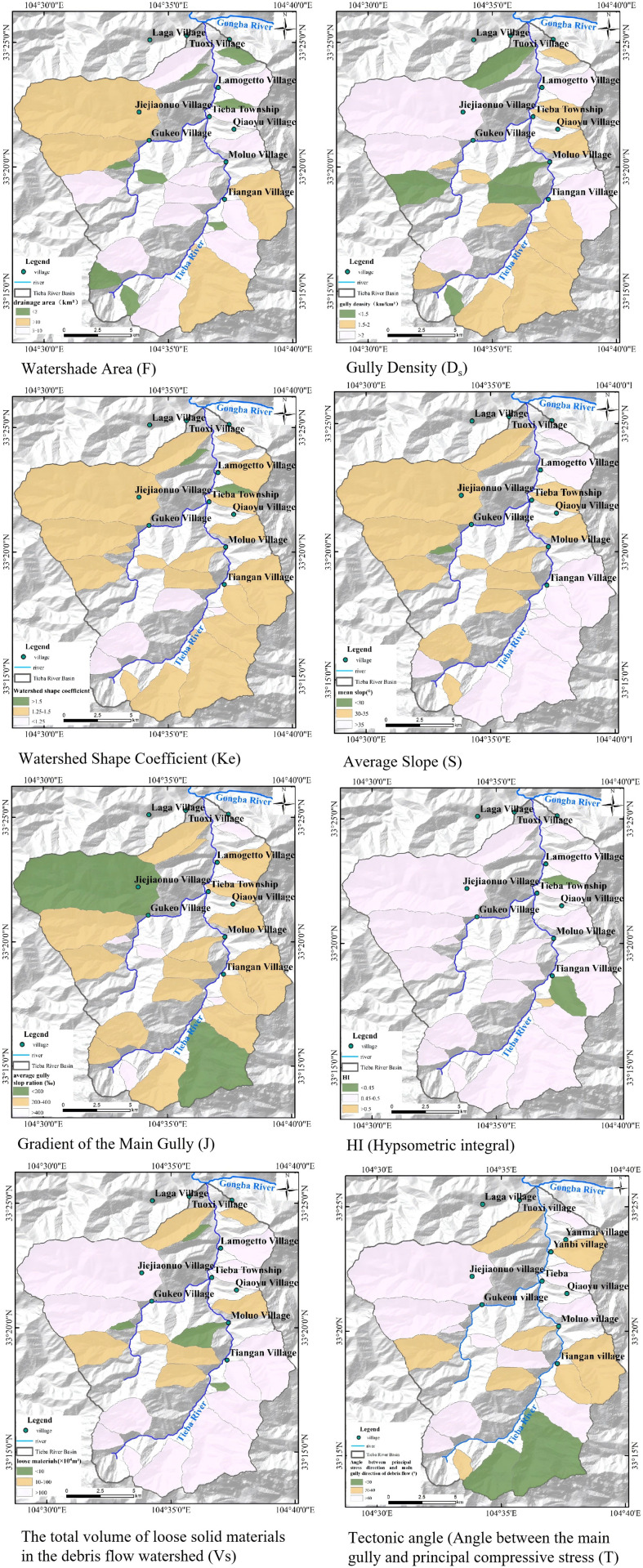
Grading maps of evaluation factors (The specific parameters of each debris flow channel are available in the dataset (https://osf.io/3kgjd/overview?view_only=a20df6c94339414bb7b62780ca618ec1). Shaded relief map of the study area derived from ALOS 12.5 m DEM data were obtained from the Geospatial Data Cloud (http://www.gscloud.cn)).

Using the 24 debris flow gullies in the Tieba River Basin as evaluation units, and drawing on relevant research findings and analysis of debris flow activity characteristics in the study area, the evaluation indicators were categorized into 3 groups comprising 8 factors, each assigned a score ranging from 1 to 3 (refer to Table 4). Standardized grading and scoring were conducted for each evaluation indicator, referring to Table 6. Graded maps for each evaluation factor were generated using the ArcGIS platform (Fig 13). Through this scoring process, all hazard-inducing factors were converted into dimensionless values suitable for direct computation, as presented in Table 5. The disaster entropy (E_j_), information utility value (V_j_), and corresponding weights (W_j_) for each evaluation indicator of the debris flow gullies were calculated using Equations 23–27, with the computational results shown in Table 6.

**Table 4:** Standardized classification of debris flow gully hazard assessment indicators.

Categories	Evaluation Indicator	Standardized Scoring		
		1	2	3
Geomorphic Factors	Watershade Area (F/km^2^)	>10	<3	3 ~ 10
	Watershed Shape Coefficient (Ke)	>1.5	1.25 ~ 1.5	<1.25
	Average Slope (S/°）	0 ~ 30	30 ~ 35	>35
	Gully Density (Ds/km/km^2^)	<1.5	1.5 ~ 2	>2
	Area-Elevation Integra(HI)	<0.45	>0.55	0.45 ~ 0.55
	Gradient of the Main Gully (J/‰)	<200	200 ~ 400	>400
Debris Flow Scale	The total volume of loose solid materials in the debris flow watershed(Vs/10^4^m^3^)	<10	10 ~ 100	>100
Tectonic Angle	Angle between the main gully and the principal compressive stress (°)	<30	30-60	>60

**Table 5:** Assignment values of assessment indicators for debris flow gullies.

Debris Flow Watershed No.	Watershed Area (F)	Gully Density (Ds)	Watershed Shape Coefficient (Ke)	Average Slope (S)	Gradient of the Main Gully (J)	HI	The Total Volume of Loose Solid Materials in the Debris Flow Watershed (Vs)	Tectonic Angle (T)
1	2	2	2	3	3	3	2	3
2	3	1	2	2	2	3	2	2
3	3	3	2	3	2	3	3	2
4	2	3	1	2	2	3	1	2
5	1	3	2	2	1	3	3	3
6	1	3	2	2	2	3	3	3
7	3	2	2	2	2	3	2	3
8	2	2	2	1	3	3	1	3
9	3	1	2	2	2	3	2	2
10	2	2	2	2	3	3	1	3
11	3	1	2	2	2	3	2	2
12	3	1	2	2	2	3	2	3
13	3	2	2	3	2	1	3	2
14	3	2	3	2	2	3	2	2
15	2	2	3	3	3	2	1	3
16	3	3	3	2	2	3	3	3
17	3	2	2	3	2	3	3	3
18	1	2	2	3	1	3	3	1
19	2	2	3	3	2	3	3	3
20	2	1	2	2	3	3	2	2
21	3	2	2	3	2	3	3	1
22	1	3	2	3	2	3	3	2
23	3	2	2	2	2	3	2	3
24	2	2	1	2	3	1	1	3

**Table 6:** Disaster entropy and weights of evaluation indicators.

Evaluation Indicator	Watershade Area (F)	Gully Density (Ds)	Watershed Shape Coefficient (Ke)	Average Slope (S)	Gradient of the Main Gully (J)	HI	The Total Volume of Loose Solid Materials in the Debris Flow Watershed (Vs)	Tectonic Angle (T)
Ej	1.9258	1.7948	1.8391	1.9528	1.8747	2.1306	1.8626	1.9947
Vj	−0.9258	−0.7948	−0.8391	−0.9528	−0.8747	−1.1306	−0.8626	−0.9947
Wj	0.1255	0.1078	0.1138	0.1292	0.1186	0.1533	0.1170	0.1349

**Fig 14:**
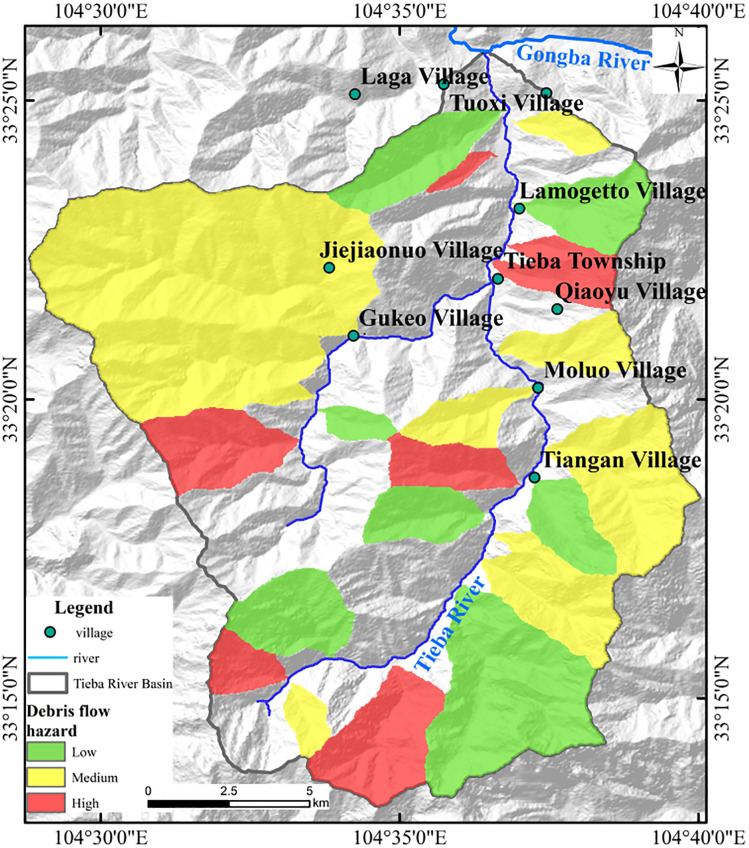
Hazard Assessment Map of the Tieba River Basin (Shaded relief map of the study area derived from ALOS 12.5 m DEM data were obtained from the Geospatial Data Cloud (http://www.gscloud.cn)).

After establishing the weights for each evaluation indicator and hazard-contributing factor, Equation 13 was utilized to calculate the hazard degree value for each debris flow gully. The results of these computations are displayed in Fig 14.

The results indicate that the hazard degree values of the 24 debris flow gullies in the study area range from 2.01 to 2.75. Utilizing the natural breaks classification method, these gullies were categorized into three hazard levels: low, medium, and high. Specifically, 8 gullies (33.3% of the total) were classified as low hazard; 10 gullies (41.7%) as medium hazard; and 6 gullies (25%) as high hazard.

## 5. Discussion

Almost all the key factors for the hazard assessment of debris flows require qualitative or quantitative analysis of the scale of debris flows. In regional assessments, geomorphic parameters such as drainage basin area, relative elevation difference, and main gully gradient are commonly used to represent the water collection capacity of debris flows and the magnitude of potential energy provided, and then indirectly characterize the scale of debris flows [42, 43]. Or geomorphic erosion stage parameters such as area and elevation are used to characterize the amount of materials that can be provided to debris flows. In numerical simulations of single gully basins, according to the form of material sources within the basin, whether they are landslide-type material sources or gully deposit-type material sources, different hydrodynamic models are used for scale quantification [44–46]. For remote sensing image data of long time series, the area change of slope material sources within the basin can be obtained, and then the sediment transport rate can be calculated for quantitative analysis. These methods have been verified in many regions and have achieved good results [47–50]. However, for the key factors controlling the scale of debris flow material sources, that is, the influence of the lithology of strata at different elevations within the basin and the tectonic conditions on the integrity of rock and soil masses, there is insufficient quantification. Moreover, the calculation data mainly focus on two-dimensional planar areas, and the quantification of the volume of material sources is relatively rare. Therefore, taking the clustered debris flow events in the Tieb River Basin as an example, based on field investigations and the analysis of basic geomorphic factors, this paper attempts to propose a prediction and calculation method for the hazard range of debris flows based on the quantification of special material sources. and further constructs a hazard assessment method for clustered debris flows in small mountainous basins considering the controlling effect of regional tectonic stress fields.

**Fig 15:**
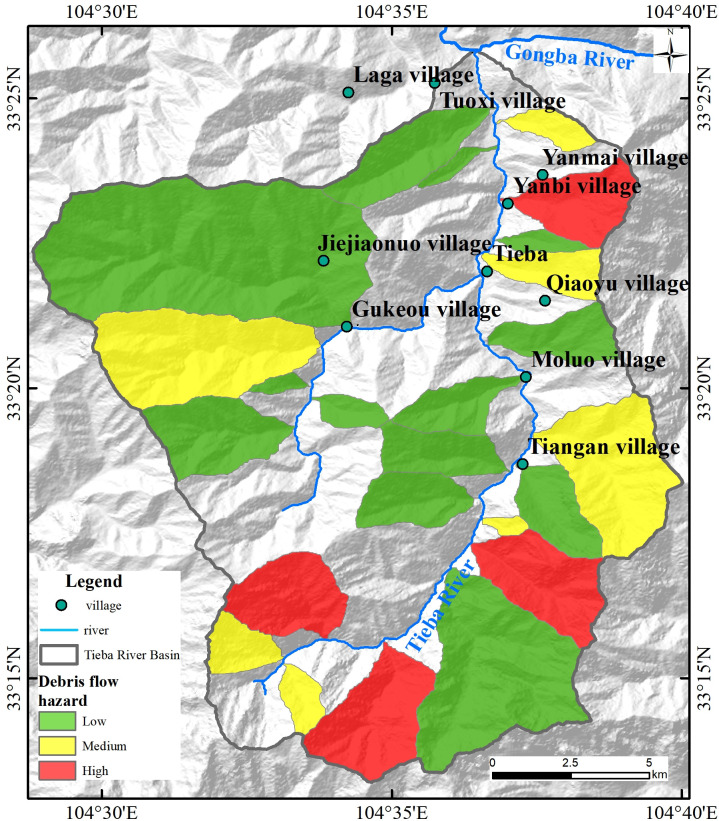
Hazard assessment map of the Tieba river basin without considering the tectonic angle (T) (shaded relief map of the study area derived from ALOS 12.5 m DEM data were obtained from the Geospatial Data Cloud (http://www.gscloud.cn)).

**Fig 16:**
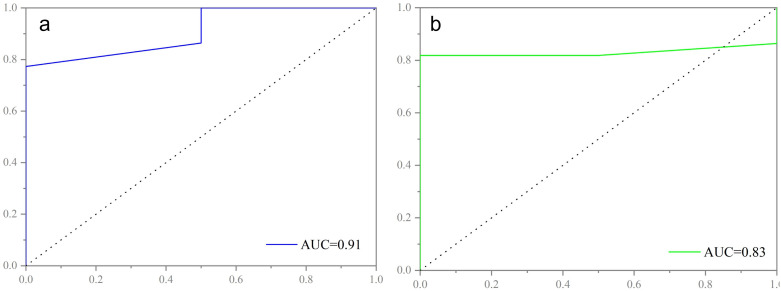
The roc curve of two results (Figure (a) presents the hazard assessment of considering the T factor, and Figure (b) presents the hazard assessment of without considering the T).

（1） Lithology dictates the weathering rate and the type of weathering products, thus exerting an influence on the initial thickness and material composition of the accumulated deposits. In contrast, the structural characteristics of the rock mass (such as bedding planes and joints) govern the weathering depth and potential slip surfaces, ultimately leading to a specific thickness distribution pattern under the combined influence of topography and water. It was discovered that the relationship between colluvium thickness and microtopography (e.g., hill noses, grooves) in shale regions is not a straightforward linear relationship; in certain locations, the thickness of residual soil and highly weathered bedrock can surpass 30 meters [51]. And geophysical methods were used to probe the thickness of colluvial deposits in high-altitude, cold regions. The results showed that their distribution is influenced by topography (such as leeward slopes). The bedrock depth is generally 4–10 meters, with some exceeding 15 meters in thickness [52]. Within the Tie River Basin, this analysis estimated loose solid material reserves in debris flow gullies. Three source types were categorized according to lithological features, and their thickness values (25 m, 2 m, and 1 m, respectively) were calculated. These were then multiplied by the planar area obtained from remote sensing interpretation. Thickness is affected by microtopography (slope curvature, gully width), and the average value is a conservative estimate. The method depends on empirical approaches and field survey accuracy. In future phases, 3D technology might be integrated for improved three-dimensional precision in thickness measurement. The influence of environmental factors like fault activity, rainfall, and temperature on thickness variations across lithological materials requires further exploration. These factors can be comprehensively examined via field surveys and geotechnical strength test results.
(2) The regional tectonic stress field (e.g., principal stress direction, number of deformation phases) provides the macroscopic background for the development of debris flows [53]. By generating fault and joint networks, tectonic stress primarily controls the development of drainage systems (e.g., rivers developing along faults). Concurrently, multi-phase tectonic impact exacerbates the fragmentation degree of rock and soil masses, providing abundant material sources for debris flows. Meanwhile, it also enhances the permeability of rock masses and reduces their stability [54, 55]. For the selection of the tectonic angle (T) in the Tieba river basin, one of the two key factors significantly impacts the accuracy of debris flow hazard assessment in small watersheds. To evaluate this, we applied the disaster entropy model while excluding the tectonic angle (T) factor for comparative analysis. The evaluation results (Fig 15) revealed 16 high and medium hazard debris flow gullies, one fewer than those identified in the model incorporating the tectonic angle (T). Notably, the Moluo Gully (Number 9, Fig 5) and the adjacent Renwu Gully (Number 12), which caused severe damage during the August 17, 2020, mass debris-flow event, were classified as low hazard in this model, a discrepancy with the actual disaster conditions. ROC curve analysis (Fig 16) demonstrated that the integrated tectonic angle model achieved higher accuracy (AUC value 0.907) compared to the unmodified model (AUC value 0.856). These comparative results highlight the advantages of incorporating the tectonic angle (T) model in hazard assessment.

(3) The study of debris flow scouring range is a core component of disaster hazard assessment. Its primary objective is to predict the movement distance, accumulation area, and affected regions of debris flows. Three main methodologies are commonly used: empirical statistical methods, numerical simulation techniques, and experimental research approaches [56–58]. The empirical statistical method establishes statistical relationships between the scouring distance, topographic parameters (such as elevation differences and channel gradients), and debris flow volume based on historical event observation data. Previous empirical prediction research on debris flow deposition dimensions in tectonically active mountain areas has revealed a prominent power-law correlation between total debris volume (Qt) and maximum runout length/width of the accumulation fan [41,59,60]. Similar power-form empirical formulas were established in the Wenchuan area disturbed by strong earthquakes [60], verifying the rationality of the statistical model in this study. Furthermore, the lithology of the Wenchuan earthquake area [60] is igneous and metamorphic rocks, which can be compared with the sedimentary rock area in this study to further analyze the applicability of the statistical formula. This approach is computationally simple and suitable for rapid regional-scale assessments. However, its effectiveness highly depends on the completeness and quality of historical data, making it difficult to apply in data-deficient regions. When fitting the formula to estimate the hazard range of debris flows, the analysis is conducted using the deposition fans of 11 debris flow gullies within the basin as samples. The relatively low prediction accuracy for width B is primarily attributed to the significant influence of local topographic incision and subsequent erosion on fan morphology, which is not adequately captured by the current set of variables-particularly due to the absence of dynamic topographic factors such as slope curvature and channel longitudinal gradient. In the next phase of research, it is recommended to incorporate parameters derived from high-resolution DEMs into the model and to conduct residual spatial autocorrelation tests separately for sub-models with R^2^ values below 0.6, in order to identify potential sources of systematic bias. Furthermore, the sample size can be expanded by including small basins with similar tectonic and lithological conditions from the northeastern margin of the Qinghai-Tibet Plateau, thereby improving the accuracy and broadening the applicability of the hazard prediction formula.

## 6. Conclusion

The text elaborated on an investigation regarding clustered debris flow disasters in the Tieba River Basin. Two key factors affecting debris flow hazards in small mountain basins were identified: the scale of debris flows (source characteristics) and tectonic impacts. Tectonic stress controls rock mass fragmentation, loose material formation, and drainage development rather than directly triggering landslides. The solid materials come from weathered unstable rocks, gully deposits, and slope colluvium instead of large-scale landslides. Through the analysis of remote sensing images, a method was put forward for the quantitative calculation of the volume of solid material sources based on lithological categories and geomorphic elevations. Moreover, a formula was established to determine the scale of debris flows in small mountainous watersheds. Additionally, the geomorphic indices of the river system direction, influenced by tectonic conditions, were quantified. Subsequently, a prediction formula for the hazard range of single debris flow ravines and a regional debris flow hazard zoning based on a disaster entropy model were implemented. The principal conclusions of the paper are as follows:

(1) Through on-site field investigations and the utilization of high-resolution satellite imagery, combined with the integration of stratigraphic lithology data, the debris flow material sources in the study area have been classified into three categories: gully deposits, potential unstable rocks, and slope material sources. Geographic Information Systems (GIS) are utilized to demarcate the planar scope of potential landslides and collapses related to debris flow material sources across the entire basin. By leveraging information regarding different stratigraphic lithologies, the thickness of landslides and collapses within the basin is estimated, enabling the calculation of the reserves of debris flow material sources in the study area. The findings suggest that the study area contains 407 × 10^4^ m^3^ of gully deposits, 2803 × 10^4^m^3^ of potential unstable rocks, and 167 × 10⁴ m^3^ of slope material sources, with a total reserve of loose materials amounting to 3377 × 10^4^ m^3^. The volume is an approximate estimate with certain uncertainty, which is more in line with the actual data characteristics.
(2) The rainstorm-flood method is employed to compute the total scale of debris flows (Q_t)_ for 24 debris flow gullies in the Tieb River Basin under a rainfall frequency with a 200-year return period. Through regression analysis, a predictive formula for the scale of debris flows is developed, which incorporates the drainage basin area, the reserves of loose material sources, the elevation difference of the basin, and the longitudinal gradient of the main gully. The formula is expressed as Q_t_ = 0.077(Vs/F)^0.818^ + 0.075J^-4.725^ + 4.93H^3.385^. Based on the measured parameters of the deposition fans, predictive formulas for the hazard range of debris flows are established: B = 0.024Q_t_^0.441^; L = 0.066Q_t_^0.299^.
(3) The river system is derived from the Digital Elevation Model (DEM) of the study area using Geographic Information System (GIS) technology. Analysis indicates that the river system distribution exhibits three primary orientations: Max1 at 6°, Max2 at 138°, and Max3 at 90°. The angle between Max1 and Max2 is chosen for calculation, determination, and it is determined that the direction of the principal stress field is NW18°. This direction is nearly parallel to the strike of the regional main fault, approximately perpendicular to the strike of the secondary fault, and exactly coincides with the dip direction of the rock strata in the high-level caving zone of the formation area at the rear edge of the debris flow small watershed. This coincidence promotes the occurrence of gravitational landslides in the rock mass.
(4) By integrating the volume of loose solid materials from debris flows, the included angle between the strike of the main gully and the direction of the regional principal stress, along with six geomorphic parameters—drainage basin area, drainage basin shape coefficient, gully density, longitudinal gradient of the main gully, average slope of the basin, and the HI (hypsometric integral) index—and using the disaster entropy model, the hazard assessment results for clustered debris flows in small basins are computed. The findings indicate that the hazard values for the 24 debris flow gullies in the study area range from 2.01 to 2.75. Among these, 16 gullies exhibit medium to high hazard, constituting 66.7% of the total, which aligns closely with actual disaster situations.
(5) This research presents a quantitative approach and model for evaluating the hazard of debris flows in the Tieb River Basin, and it also provides a reference for the risk assessment and management of debris flows.
